# Inductive effects in amino acids and peptides: Ionization constants and tryptophan fluorescence

**DOI:** 10.1016/j.bbrep.2020.100802

**Published:** 2020-09-13

**Authors:** Jesús Lara-Popoca, Henrik S. Thoke, Roberto P. Stock, Enrique Rudino-Pinera, Luis A. Bagatolli

**Affiliations:** a Instituto de Biotecnología, Universidad Nacional Autónoma de México, Departamento de Medicina Molecular y Bioprocesos, Av. Universidad #2001, Col. Chamilpa, 62210, Cuernavaca, Morelos, Mexico; b MEMPHYS - International and Interdisciplinary Research Network, Odense, Denmark; c Instituto de Investigación Médica Mercedes y Martín Ferreyra (INIMEC-CONICET-Universidad Nacional de Córdoba), Friuli 2434, 5016, Córdoba, Argentina

**Keywords:** Inductive effects, Amino acids, Peptides, Ionization constants, Tryptophan fluorescence, Computational model

## Abstract

Although inductive effects in organic compounds are known to influence chemical properties such as ionization constants, their specific contribution to the properties/behavior of amino acids and functional groups in peptides remains largely unexplored. In this study we developed a computationally economical algorithm for *ab initio* calculation of the magnitude of inductive effects for non-aromatic molecules. The value obtained by the algorithm is called the *Inductive Index* and we observed a high correlation (R^2^ = 0.9427) between our calculations and the pK_a_ values of the alpha-amino groups of amino acids with non-aromatic side-chains. Using a series of modified amino acids, we also found similarly high correlations (R^2^ > 0.9600) between Inductive Indexes and two wholly independent chemical properties: i) the pK_a_ values of ionizable side-chains and, ii) the fluorescence response of the indole group of tryptophan. After assessing the applicability of the method of calculation at the amino acid level, we extended our study to tryptophan-containing peptides and established that inductive contributions of neighboring side-chains are transmitted through peptide bonds. We discuss possible contributions to the study of proteins.

## Highlights

• An algorithm for *ab initio* calculation of inductive effects in amino acids and peptides was developed.
• The inductive index calculations correlate closely with ionization behavior.
• The inductive index calculations correlate closely with tryptophan fluorescence.
• Inductive effects may impact the properties of functional groups in proteins.

## 1 Introduction

Inductive effects are defined as the polarization of a bond by electronegativity differentials between the atoms of adjacent bonds. These effects propagate through the bond structure of the molecule and are distinct from electrostatic or field effects, which are by definition not transmitted through the chemical bonds. Inductive effects were described in detail, albeit empirically, already in the 1930's and what was then established was incorporated into advanced organic chemistry textbooks [1]. Inductive effects have been long recognized to play a central role in the ionization of chemical groups and in many other chemical properties. Although the measurement, and more importantly, prediction of ionization constants of functional groups in peptides and proteins is an essential aspect of protein research, the importance of inductive effects remains largely unexplored.

The development of formal methods to calculate the magnitude of inductive effects was attempted most notably in the classical works of Hammett [[2], [3], [4]] and Taft [[5], [6], [7]]. Taft studied the variation of acid dissociation constants of simple non-aromatic carboxylic acids and amines substituted with different atoms. These careful studies established that electron-withdrawing substituents had the effect of favoring acid dissociation (lowering pK_a_) of the reporter groups even in non-aromatic (non-resonant) molecules. Taft's investigations were semiempirical, that is, they could not provide an *ab initio* method of calculation of the magnitude of the effect on the measured property but they established two general features of inductive effects: i) they depend on the electronegativity of the substituent and ii) they depend on its distance -in number of intervening bonds-from the group being measured. Hammett had applied a similar outlook to the study of some aromatic molecules, most particularly substituted benzoic acids. He demonstrated that substituents of varying electronegativity influenced both the ionization constants and reactivities of the reporter groups in a manner much like the one described by Taft, although the relatively simple concept of distance (in number of bonds from the reporter group) necessarily incorporated the notion of position of the substituent in the phenyl ring due to the resonance or mesomeric effect.

A significant step was taken in the early 1960's, when a general *ab initio* method for calculating the magnitude of inductive effects in non-aromatic ionizable molecules of, in principle, any size, was reported by Chiang and Tai [8]. Their calculation method took into account the electronegativity of all atoms in the molecule (in the Pauling scale), bond lengths, formal charges and the number of intervening bonds between the polarizing bond and the (inductively) polarized bond under study (e.g., the O-H bond in a carboxylic acid). They called the number provided by their method the *Inductive Index* and tested it in a very large number of molecules and chemical properties, finding a very good agreement between their calculations and the measured chemical parameters. Since then, however, the concept of electronegativity in its relation to the state of hybridization of the atoms in the bond has been much improved [9,10] introducing the possibility of further refinement of Chiang and Tai's work.

From a theoretical perspective, all α-amino acids fit the general scheme of the classic works of Taft and Hammett, that is, they all have an identical basic structure (the backbone) and a substituent (the side-chain) which confers to each its chemical identity. Therefore, if inductive effects are relevant at this level of description, it is not unreasonable to expect that differences in the ionization of the α-carboxyl and α-amino groups of amino acids are a function of the inductive contributions -as approached by Chiang and Tai-of the side-chain groups. In fact, if this is true, there should exist a direct correlation between the ionization constants of the α-carboxyl and α-amino groups of α-amino acids with *non-ionizable side-chains,* i.e. the side-chain does not ionize in the pH interval between the ionizations of the α-carboxyl (~2) and the α-amino groups (~9). This correlation would not be expected in pK_a_ values of the α-groups of those amino acids in which the side-chain ionizes -and therefore its inductive contribution changes-between both α-group ionizations. However, in the course of our research we found a surprising level of inconsistency in the pK_a_ values reported through the years, probably reflecting different experimental conditions used to measure them (titrants, amino acid concentration) and methods of calculation [[11], [12], [13], [14]].

In the case of ionizable side-chains, the same reasoning should hold true: their pK_a_ values should respond to the inductive contribution of the rest of the molecule. This includes the effects of both the state of protonation of the α-groups and their modification by, for example, amidation, esterification, acetylation or even peptide bond formation, which should alter their inductive contributions. This behavior should also extend to side-chains in peptides, that is, specific inductive contributions of formal charges present in terminal α-groups and, more generally, to the contribution of neighboring residues with particular side-chains, to the chemical properties of any given side-chain in a sequence.

Since the pioneering work of Gregorio Weber on the fluorescent properties of aromatic amino acids, most notably tryptophan and tyrosine [15], it has been known that their fluorescence closely follows the state of ionization of the α-groups. It increases when these groups deprotonate and decreases when they protonate so closely that their ionization constants can be determined from changes in fluorescence intensity upon titration [16]. The mechanisms proposed, however, have not generally given careful consideration to inductive effects, save passing references [[17], [18], [19]]. When the fluorescent group is part of a peptide, similar effects of ionization on fluorescence have been reported. In glycyl-tryptophan peptides in which the terminal amino group is progressively removed from the tryptophan by addition of an increasing number of glycyl residues two things are apparent: First, the effect of protonation/deprotonation of the terminal amino group on fluorescence intensity decreases with distance. Second, the change in fluorescence that accompanies terminal amino group deprotonation is considerably smaller and follows the altered pK_a_ of the terminal group [20]. In this particular reported case, the effect of a progressively more removed electron withdrawing group (a protonated amino) was studied by the authors. Although field effects have been generally favored as an explanation for these observations, an inductive interpretation as described above is also consistent with the data. Inductive effects, albeit less marked, should also be exerted by side-chains with different inductive contributions on neighboring amino acids. In other words, changing the amino acid adjacent to the tryptophan with a different residue -all other things equal-should be reflected in its fluorescence properties due to changes in inductive contributions.

In this study we endeavored to: i) refine the method of *ab initio* calculation of Inductive Indexes originally proposed by Chiang and Tai to extend it to amino acids and peptides; ii) correlate the calculated values with measured differences in the pK_a_ of ionizable groups of amino acids and some modified derivatives; iii) establish whether the results of the calculations correlate adequately with differences in fluorescence properties of the indole group in tryptophan and some model peptides containing tryptophan. The implications of our findings are discussed in the context of the current understanding of peptides and proteins.

## 2 Materials and methods

### 2.1 Amino acids and potentiometric titration reagents

All standard l-amino acids were analytical standards from Sigma-Aldrich. Modified amino acids were the highest available purity from Sigma-Aldrich and Research Organics. Water was twice-distilled crystallography grade. Titrants (0.1 N NaOH or 0.1 N HCl) were standardized solutions from JT Baker. Standard pH buffers for electrode calibration from Hannah (HI 6000 series: pH 1.679, pH 4.010, pH 6.862, pH 7.010, pH 10.010).

### 2.2 Peptides and reagents for fluorescence measurements

Peptides were obtained from JPT Peptide Technologies (Berlin, Germany) and were of a purity > 95%. All peptides had Trp as fluorophore, the N terminus acetylated and the C terminus amidated. The tetrapeptides had Gly as the linking amino acid.

### 2.3 Determination of ionization constants of α-groups and ionizable side-chains of unmodified and modified amino acids by potentiometry

Titrations were conducted under rigorously identical conditions in a Metrohm 853 automatic titration unit equipped with a water jacketed titration vessel for temperature control (25 °C ± 0.1 °C), magnetic stirring and a Metrohm Biotrode pH electrode. Initial volume was 10 ml in all cases; titrations were performed under a stream of humid nitrogen and using a CO_2_ trap. Electrode drift was stabilized to <0.5 mV/min and the electrode was calibrated each day with five buffer standards. For titration of α-amino groups and ionizable side-chains, amino acids were initially dissolved at 20 mM in 0.1 N HCl and then titrated with 0.1 N NaOH; ionization constants (expressed as pK_a_) were determined by analysis of the titration curve by standard second derivative calculations implemented in the software supplied with the titration system. In the case of ionization constants of α-carboxyl groups, amino acids were initially dissolved in distilled water at 20 mM and titrated by sequential addition of 0.1 equivalents of titrant, in this case 0.1 N HCl. The experimental methods and calculations were performed as described in detail in Albert and Serjeant [21]. All titrations were replicated at least three times on different days and with two independent batches for most standard amino acids.

### 2.4 Fluorescence measurements

Fluorescence lifetime measurements were performed in a Chronos ISS (Champaign, IL, USA) Multifrequency phase shift and modulation fluorimeter, using a 280 nm LED (ISS model 90101) for excitation and p-terphenyl in EtOH (τ = 1.05 ns) as a reference. To eliminate scatter from excitation light the fluorescence emission was collected through a WG320 long-pass filter. Data were collected until the standard deviation from each measurement of phase delay (ϕ) and modulation (M) were at most 0.2° and 0.004 respectively. Diode modulation frequency was ranged logarithmically from below to above the crossover of ϕ and modulation M in at least 8 steps (usually between 20 MHz and 200 MHz), and analyzed for each peptide using the Vinci software (ISS) assuming a single lifetime with a Lorentzian distribution (both lifetime and width being variables). χ^2^ scores were all below 1.40 and generally below 1 (see Table S3). A special case is given for tryptophan amide which was the only compound which showed two lifetimes (τ = 1.44 ns with a fraction of 0.71 and τ = 6.94 ns with a fraction of 0.29). For further analysis, the lifetime of τ = 1.44 ns was used, as this was the primary contribution. Similarly, a single lifetime was used for tryptophan as analysis using two or more lifetimes showed no improvement in the score, and fractions of other lifetimes were negligible. All measurements where done using water of Milli-Q quality at a temperature of 25 °C ± 0.1 °C, pH of 6.81.

### 2.5 Calculation of inductive indexes

#### 2.5.1 Chiang and Tai's algorithm

The original algorithm depends on three parameters: the electronegativity (in the Pauling scale) of all atoms involved in the calculation, the bond lengths between them and an arbitrary parameter 1/α called the transmission factor (see below). For a hypothetical molecule.

**Figure:**
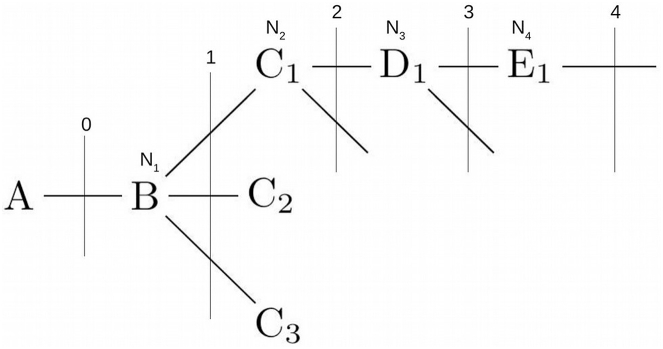

Chiang and Tai defined the *polarity index* (*δ*_*AB*_) of the bond A-B as the weighed difference between the electronegativities (*χ*) of the atoms A and B as $\delta_{AB}=\frac{\chi_{B}-\chi_{A}}{\chi_{B}+\chi_{A}}$ .

They also defined the *intensity of polarity* as the polarity index divided by the bond distance ($r_{AB}$ in Å), $\frac{\delta_{AB}}{r_{AB}}$.

The *Inductive Index* on atom A is defined as the sum of three contributions: a) *i*_*0*_, the effect of the immediate atom (or atoms in non-terminal bonds) bonded to atom A, b) *i*, the aggregated effect of all atoms not directly bonded to atom A, where the effect of their intensity of polarity is attenuated by their distance (the *bond order, n*) using the parameter ${\left(\frac{1}{\alpha }\right)}^{n}$ (see below) and, c) *i*_±_, the effects of any formal charges present in the molecule, also attenuated by ${\left(\frac{1}{\alpha }\right)}^{n}$.

Formally, then, the *Chiang and Tai* *Inductive Index (I_A_)* on atom A is defined by equation (1) as

(1) *I*_*A*_ = i_0_ + i + i_*±*_

Where $i_{0}=\frac{\delta_{AB}}{r_{AB}}$.

Equation (2) shows the contribution of all the bonds of order 1 or higher.

(2) $$i=\frac{1}{\alpha }\sum {\left(\frac{\delta }{r}\right)}_{1}+{\left(\frac{1}{\alpha }\right)}^{2}\sum {\left(\frac{\delta }{r}\right)}_{2}+{\left(\frac{1}{\alpha }\right)}^{3}\sum {\left(\frac{\delta }{r}\right)}_{3}+\;...\;+{\left(\frac{1}{\alpha }\right)}^{n}\sum {\left(\frac{\delta }{r}\right)}_{n}$$

where $\frac{1}{\alpha }$ is called the *transmission factor* and α is equal to 2.7; *n* is the *bond order*, that is, the distance in terms of covalent bonds from A (for example, B-C_*n*_ all have order 1, C_1_-D_1_ has order 2 and A-B has order zero), $\sum {\left(\frac{\delta }{r}\right)}_{n}$ is the sum of all the $\frac{\delta_{XY}}{r_{XY}}$ of order *n*.

Finally, equation (3) defines the sum of the contributions of formal charges as

(3) $$i_{\pm }={\left(\frac{\pm N_{1}}{r_{\pm }}\right)}_{0}+\frac{1}{\alpha }\sum {\left(\frac{\pm N_{2}}{r_{\pm }}\right)}_{1}+{\left(\frac{1}{\alpha }\right)}^{2}\sum {\left(\frac{\pm N_{3}}{r_{\pm }}\right)}_{2}+{\left(\frac{1}{\alpha }\right)}^{3}\sum {\left(\frac{\pm N_{4}}{r_{\pm }}\right)}_{3}+\;\ldots \;+{\left(\frac{1}{\alpha }\right)}^{n}\sum {\left(\frac{\pm N_{n}}{r_{\pm }}\right)}_{n}$$

where *r*_±_ is the bond length in the ionized group, and ±*N*_*i*_ can be equal to 1, -1 or 0.

#### 2.5.2 Modified CT algorithm (MCT)

Our method of calculation modified Chiang and Tai's algorithm in several ways. First, we did not include bond distances in the calculations. Second, while we maintained the concept of *polarity index* (*δ*), we used values of electronegativity not of atoms in their ground state, but as they appear in molecules, that is, considering their valence state based on the concept of *orbital electronegativity* as developed by Hinze and Jaffé [9], which, unlike the Pauling scale of electronegativity, is contemporary to the publication of Chiang and Tai's opus. Third, the transmission factor $\left(\frac{1}{\alpha }\right)$ was 0.5263 (α = 1.9). Fourth, formal charges were uniform for all calculations (amino acids and peptides) but were not unitary: positive charges had a value of +1.2 and negative charges of -0.9 (see Supplementary Information for a detailed discussion of value selection). Thus, the final expression of inductive index (*ii*), or MCT, is as follows:

*ii* = i_0_ + i + i_*±*_

Where

$$i_{0}=\delta_{AB}\text{,}$$

$$i=\frac{1}{\alpha }\sum {\left(\delta \right)}_{1}+{\left(\frac{1}{\alpha }\right)}^{2}\sum {\left(\delta \right)}_{2}+{\left(\frac{1}{\alpha }\right)}^{3}\sum {\left(\delta \right)}_{3}+\;...\;+{\left(\frac{1}{\alpha }\right)}^{n}\sum {\left(\delta \right)}_{n}\text{,}$$

and

$$i_{\pm }={\left(\pm N_{1}\right)}_{0}+\frac{1}{\alpha }\sum {\left(\pm N_{2}\right)}_{1}+{\left(\frac{1}{\alpha }\right)}^{2}\sum {\left(\pm N_{3}\right)}_{2}+{\left(\frac{1}{\alpha }\right)}^{3}\sum {\left(N_{4}\right)}_{3}+\;\ldots \;+{\left(\frac{1}{\alpha }\right)}^{n}\sum {\left(\pm N_{n}\right)}_{n}\text{.}$$

Initial calculations of inductive indexes, graphs and linear regressions for individual amino acids were done using the LibreOffice Calc spreadsheet. Both graphs and regressions were later repeated using QtiPlot (GPLv2). The Inductive Index calculated was always that exerted on the C_α_ of the amino acid under scrutiny. When examining ionization of α-NH_3+_ groups, the value was calculated by the structure of the side-chain alone, considering that α-COOH is already deprotonated and, therefore, its inductive contribution is fixed for all α-amino acids. When examining the behavior of ionizable side-chains, the inductive index on C_α_ calculated was that exerted by the terminal α-groups, whether ionized or covalently modified.

Calculations of inductive index on the C_α_ of Trp in peptides incorporate: i) the contributions of all backbone elements in the chain; ii) the contribution of all side-chains except the Trp side-chain, where the state of ionization of ionizable side-chains is specified, and; iii) terminal groups, whose state of ionization or covalent modification (N-acetylation, C-amidation) is specified as well. All values calculated in the appropriate bond order with respect to the corresponding C_α_ (See Supplementary Information).

## 3 Results

### 3.1 Inductive index and ionization constants

If inductive effects of the side-chains are indeed the main factor determining the differences in the ionization constants (as pK_a_) of ionizable α-groups of α-amino acids, then it is necessarily the case that there must be a positive correlation between the pK_a_ values of the α-amino and α-carboxyl groups of all twelve α-amino acids with non-ionizable side-chains. To test this prediction, we carefully and consistently determined their pK_a_ values, which are summarized in Table 1.

**Table 1:** Measured pK_a_ values of α-amino and α-carboxyl groups of all twelve α-amino acids with non-ionizable side chains (±standard deviation, SD). All pK_a_ values were determined using the same equipment, reagents and procedures as detailed in Material and Methods.

Amino Acid	α-NH_3_^+^	α-COOH
Ala	9.80 ± 0.02	2.40 ± 0.04
Val	9.55 ± 0.01	2.33 ± 0.05
Gly	9.68 ± 0.01	2.40 ± 0.04
Ile	9.69 ± 0.01	2.38 ± 0.04
Leu	9.64 ± 0.01	2.35 ± 0.04
Gln	9.15 ± 0.01	2.22 ± 0.05
Met	9.15 ± 0.02	2.24 ± 0.05
Ser	9.11 ± 0.02	2.25 ± 0.05
Thr	9.01 ± 0.01	2.23 ± 0.05
Asn	8.80 ± 0.02	2.19 ± 0.05
Phe	9.15 ± 0.03	2.23 ± 0.05
Trp	9.42 ± 0.02	2.35 ± 0.04

**Fig. 1:**
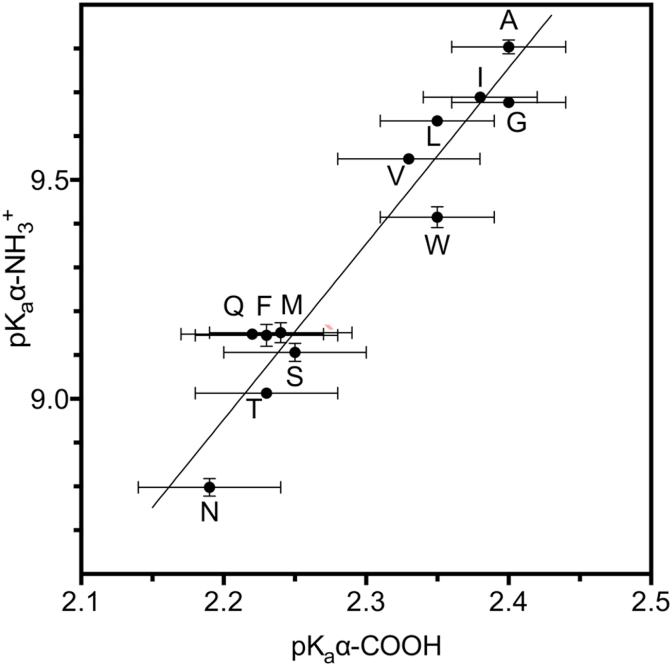
Plot of pK_a_ (±SD) values of α-amino (ordinate) against α-carboxyl (abscissa) groups for all α-amino acids with non-ionizable side-chains and linear regression line. The larger error bars of the pK_a_ values of the α-carboxyl groups are a consequence of the greater concentration of hydrogen ions (and thus total ionic strength) at the point of ionization (discussed at length in Albert and Sergeant [21], whose method we followed).

Using this self-consistent dataset of ionization constants we found a very significant correlation (R^2^ = 0.9320) between ionization of the α-amino and α-carboxyl groups in each amino acid, as shown in Fig. 1.

The outstanding correlation between the pK_a_ values of α-groups of amino acids lent tentative support to an explanation considering inductive effects as a primary cause.

In fact, if these are not considered there is no hint of an explanation of why this proportionality should exist at all (or why there are differences in the ionization constants of α-groups in the first place). We then proceeded to calculate Inductive Index values (as a measure of the magnitude of inductive effects exerted on the C_α_ by the side-chain) for all non-aromatic α-amino acids using our modified Chiang and Tai algorithm (MCT). The results are shown in Table 2.

**Table 2:** Inductive index (*ii*) values calculated for each side-chain on the C_α_ of all 15 non-aromatic α-amino acids and experimentally determined pK_a_ values of their α-NH_3+ groups (ranked from highest to lowest). The_ pK_a_ values of non-ionizable α-amino groups are the same as in Table 1 and are included for comparison. The state of ionizable side-chain groups (used for calculations) are indicated in parentheses.

Side chain	*ii* of side-chain on C_α_ (x 10^3^)	α-NH_3_^+^ pK_a_
Cys (thiol ionized)	−339.6	10.33 ± 0.02
Asp (β-carboxyl ionized)	−162.5	9.76 ± 0.02
Ala	−134.0	9.80 ± 0.02
Val	−127.1	9.55 ± 0.01
Ile	−96.8	9.69 ± 0.01
Glu (γ-carboxyl ionized)	−91.3	9.74 ± 0.01
Leu	−72.8	9.64 ± 0.01
Gly	−53.2	9.68 ± 0.01
Met	19.7	9.15 ± 0.02
Arg (guanidinium ionized)	46.5	9.08 ± 0.01
Lys (ε-amino ionized)	62.8	9.16 ± 0.01
Ser	81.5	9.11 ± 0.02
Gln	89.3	9.15 ± 0.01
Thr	92.3	9.01 ± 0.01
Asn	180.8	8.80 ± 0.02

**Fig. 2:**
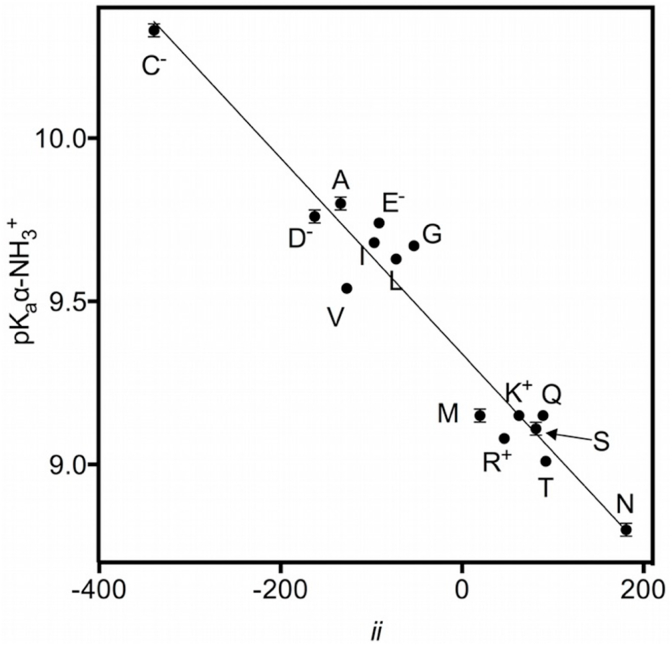
Plot of the pK_a_ (±SD) values of the α-amino groups of all non-aromatic α-amino acids against calculated Inductive Index value (*ii*, on C_α_). In the case of amino acids with ionizable side-chains, the state of ionization of the side-chain (as considered for calculation of *ii*) at the pK_a_ of the α-amino group is indicated.

When the calculated values are plotted against the measured pK_a_ values of the α-amino a significant correlation is evident (R^2^ = 0.9427, Fig. 2), with the most electron-donating side-chain (ionized Cys) resulting in the highest α-amino pK_a_ and the least electron donating one in the lowest (Asn). We chose to use the pK_a_ values of the amino groups for the correlation because they are measured more accurately due to the constraints imposed by total ionic strength and pH on potentiometric titrations at low pH. However, it is clear that since Inductive Index values correlate with α-amino pK_a_ they must also correlate to α-carboxyl pK_a_ (Fig. 1).

We then explored whether Inductive Indexes correlated with the pK_a_ values of ionizable side-chains. To this end, we determined the pK_a_ of the side-chains of four ionizable amino acids (Asp, Glu, His and Cys) with and without α-group modifications. N-acetylation was used to block the electron-withdrawing effect of protonated α-amino groups, C-amidation and C-esterification were used to remove the electron-donating effect of deprotonated α-carboxyl groups, and amino acids with both ionizable α-groups blocked were used to test the effect of removal of both charges on the pK_a_ of the side-chain. The Inductive Index calculations incorporated the structures of the covalent modifications. The results of the calculations and measurement of side-chain pK_a_ values are listed in Table 3.

**Table 3:** Inductive index values (*ii*) calculated on the C_α_ of unmodified and modified amino acids with ionizable side-chains and Trp, measured pK_a_ values of side-chain groups (Asp, Cys, Glu and His) and Mean Fluorescence Lifetime of tryptophan (τ, data for Fig. 4). N-Ac indicates α-amino acetylation. C-NH_2_ and C-OMe indicate α-carboxyl amidation and methyl esterification, respectively. Doubly modified amino acids are indicated as a combination of modifications. In the case of doubly modified His, the α-carboxyl group was N-methyl amidated (C-NHMe). When an α-group was not modified, it was assumed to be charged at the pH of deprotonation of the side-chain groups, indicated in parentheses. Note that the calculated Inductive Indexes on C_α_ do not include the side-chain itself, therefore, they are identical for all identical modifications and in the zwitterion form. (−) indicates modification not available for that amino acid.

		Side chain pK_a_				τ (ns) of Trp
α-Groups	*ii* on C_α_	Asp	Glu	His	Cys	
Zwitterion	653.0	3.81 ± 0.02	4.27 ± 0.01	6.02 ± 0.02	8.27 ± 0.01	2.55
N-Ac (α-COO^-^)	−338.2	4.72 ± 0.01	4.83 ± 0.01	6.98 ± 0.01	9.66 ± 0.01	4.24
C-NH_2_ (α-NH_3_^+^)	1305.2	2.94 ± 0.02	3.88 ± 0.01	5.35 ± 0.01	–	1.44
C-OMe (α-NH_3_^+^)	1385.7	–	–	5.26 ± 0.01	6.58 ± 0.01	–
N-Ac/C-NH_2_	314.0	4.01 ± 0.01	4.34 ± 0.02	–	–	2.71
N-Ac/C-OMe	394.6	–	–	–	8.78 ± 0.01	–
N-Ac/C-NHMe	304.9	–	–	6.42 ± 0.01	–	–

**Fig. 3:**
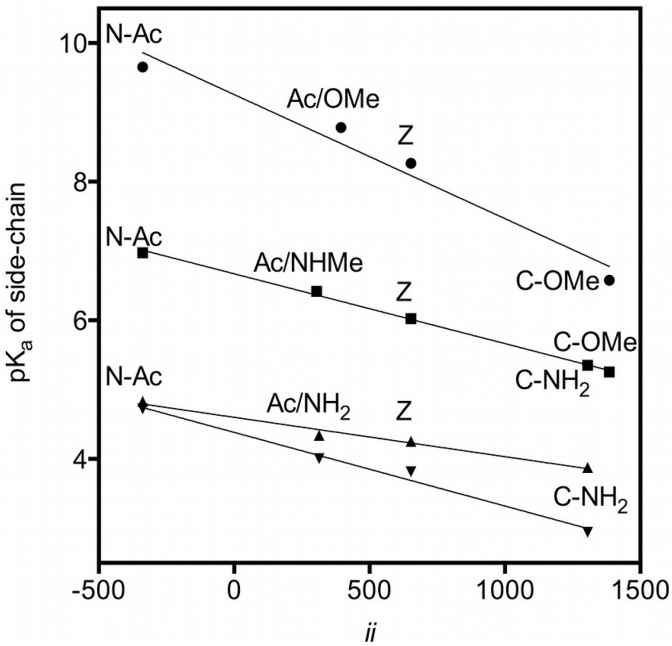
Measured side-chain pK_a_ (±SD) against calculated Inductive Index value (*ii*) on the C_α_ of each modified or zwitterionic amino acid: Cys (circles), His (squares), Glu (triangles) and Asp (inverted triangles). Z is the unmodified amino acid in its zwitterionic form and N-Ac indicates α-amino acetylation. C-NH_2_ and C-OMe indicate α-carboxyl amidation and methyl esterification, respectively. Doubly modified amino acids are indicated as a combination of modifications (Ac/NH_2_, Ac/OMe). In the case of doubly modified His, the α-carboxyl group was N-methyl amidated (Ac/NHMe).

A plot of measured side-chain pK_a_ values against calculated Inductive Index on the C_α_ of each modified or zwitterionic amino acid is shown in Fig. 3.

**Fig. 4:**
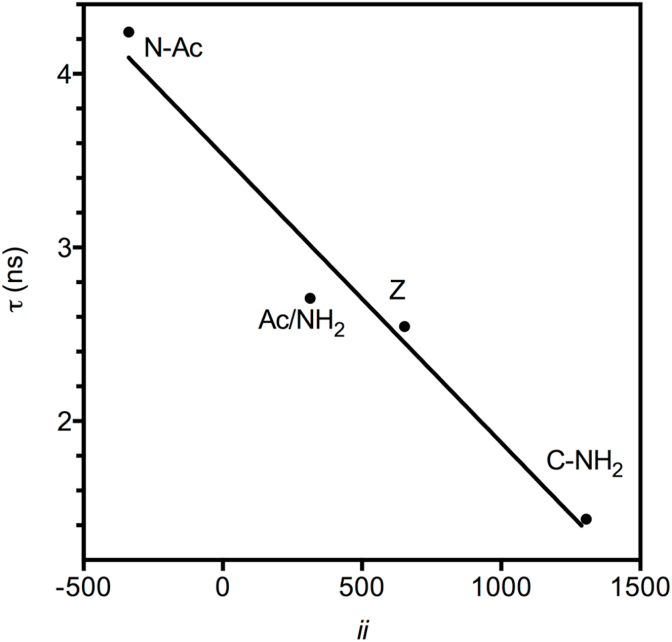
Correlation of Mean Fluorescence Lifetime (τ) of the indole group of Trp with Inductive Index (*ii*) values for the zwitterion (Z) and covalently modified α-groups (R^2^ = 0.9678). Modifications, as well as *ii* and lifetime values are those listed in Table 3. N-Ac indicates N-acetyl-Trp, C-NH_2_ indicates Trp-amide and Ac/NH_2_ indicates N-acetyl-Trp-amide (NATA).

Several things are noteworthy. First, the linear regressions show very significant correlations between calculated *ii* and measured pK_a_ values: His (R^2^ = 0.9978), Asp (R^2^ = 0.9869), Glu (R^2^ = 0.9802) and Cys (R^2^ = 0.9670). Second, removal of the positive charge in the α-amino group by N-acetylation results in the greatest side-chain pK_a_ in all amino acids, and removal of the negative charge in the α-carboxyl group in the lowest, with the zwitterion and the doubly modified amino acids giving intermediate values, with the zwitterion giving consistently a slightly lower pK_a_ than the doubly-modified amino acid. Third, the effect of the modifications is variable, that is, the change in pK_a_ with *ii* value (the slope) varies for each amino acid side-chain. Fourth, there is a difference in slope between the β-carboxyl group of Asp and the γ-carboxyl group of Glu (see Discussion). Finally, in the case of His, where two different modifications of the α-carboxyl group were available (amidation and methyl esterification), the calculation of Inductive Index discriminates between the two values of measured pK_a_. These points will be taken up in more detail in the Discussion.

### 3.2 Inductive index and tryptophan fluorescence

Fluorescence of the indole group of tryptophan is known to be strongly affected by the state of protonation of its α-groups. Its intensity increases with increasing pH during titration as each of the α-groups deprotonate. We used the same covalent α-group modifications in tryptophan to examine the correlation between Inductive Indexes and Trp fluorescence as Mean Fluorescence Lifetime (denoted by τ). We chose to measure fluorescence lifetime because it is a more robust measure than fluorescence intensity alone, as it is independent of fluorophore concentration. Measured values are listed in the last column of Table 3 and a plot of τ against Inductive Index is shown in Fig. 4.

Our next step was to examine whether inductive effects, as calculated by us, propagate through peptide bonds to a neighboring amino acid. To this end we examined a series of peptides containing tryptophan. We calculated the Inductive Index value on the C_α_ of the Trp residue of each peptide and measured their fluorescence (as τ). The values are listed in Table 4.

**Table 4:** Calculated inductive index values on the C_α_ of the single tryptophan in peptides and Mean Fluorescence Lifetime values (τ). All peptides were terminally blocked by N-acetylation and C-amidation. The negative charge on the aspartic acid residue indicates that all measurements were performed well above the pK_a_ of its β-carboxyl group.

Peptide	*ii* on C_α_ of Trp (x 10^3^)	τ (ns)
WN	−7.8	1.55
WM	−31.3	1.71
WV	−52.7	1.91
WA	−53.7	1.82
GGWD^-^	229.9	1.08
GWGD^-^	164.5	1.26
WGGD^-^	−86.1	1.80
GGWN	279.9	0.95
GWGN	171.8	1.21
WGGN	−85.1	1.80
GGWG	245.8	1.21

**Fig. 5:**
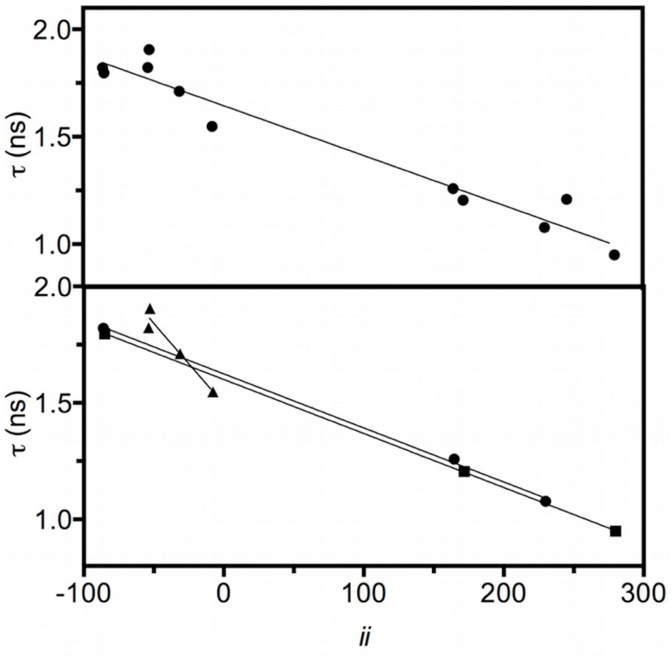
Correlation between fluorescence lifetime of all peptides and *ii* (R^2^ = 0.9498). The correlation increases when groups of related peptides are analyzed separately, that is, the Asp-containing peptides (circles, R^2^ = 0.9985), the Asn-containing peptides (squares, R^2^ = 1) and the dipeptides (triangles, R^2^ = 0.9423).

Two sets of peptides were used: the first consisted of dipeptides in which we varied the amino acid adjacent to the Trp residue (Asn, Val, Met and Ala). The second set consisted of tetrapeptides in which the distance of either aspartic acid (a strongly electron-donating residue when ionized) or asparagine (a much weaker electron donor) was varied by increasing the number of intervening glycyl residues. The plot of Mean Fluorescence Lifetime against inductive index of all peptides is shown in Fig. 5, top panel.

## 4 Discussion

### 4.1 Inductive index and the behavior of ionizable groups of amino acids

Surprisingly (to us), reported pK_a_ data for many molecules -including amino acids-are quite inconsistent across standard reference sources [22], even when reportedly obtained at the same temperature and in comparable experimental setups [[11], [12], [13], [14]]. Therefore, when we decided to explore the relevance of inductive polarization of bonds on the behavior of ionizable groups of amino acids, we found ourselves in need of obtaining a consistent dataset of pK_a_ values to work with. Obtaining the data consistently involved an experimental component and a calculational one. Experimentally, we made use of carefully controlled conditions, electrodes and reagents for potentiometric titration of ionizable groups. For calculational consistency, we avoided making any suppositions about the samples, staying well below the limits of ionic strength that would be likely to exert a measurable influence on the ionization of the measured groups [21].

α-amino acids are well suited to an approach such as the classical one originally developed by Hammett: they have a constant structure with two ionizable sites (the α-amino and α-carboxyl groups) and a variable site (equivalent to Hammett's “substituent”), namely, the side-chain. Therefore, a straightforward consequence of the effect of polarization (induction) by a non-ionizable side-chain on the ionizable α-groups is that it should affect both of them equally, that is, an electron-withdrawing side-chain should lower the pK_a_ of both ionizable α-groups, and an electron-donating one should do the opposite. Fig. 1 shows that this is indeed the case for all α-amino acids with non-ionizable side-chains: those with more acidic α-carboxyls have proportionally more acidic α-amino groups. This correlation has not been hitherto apparent from reported values in the literature, probably due to the inconsistencies mentioned above. Using four independent references correlation (as R^2^) between the pK_a_ values of α-amino and α-carboxyl groups are 0.42 [14], 0.64 [12], 0.697 [11] and 0.72 [13].

A corollary is that side-chains that are in a different chemical state (protonated/deprotonated) between α-carboxyl and α-amino ionizations would be expected to exert different inductive contributions for each chemical state, and therefore result in non-proportional pK_a_ values. This is indeed the case, as illustrated by Cys (α-carboxyl pK_a_ of ~1.8 -one of the lowest- and α-amino pK_a_ of ~10.3, the highest) but also for aspartic acid, glutamic acid and histidine.

While inductive effects have been understood, or at least considered, for small organic molecules for a long time, it is remarkable that they have not, to the best of our knowledge, been incorporated into the mainstream of current thinking about amino acids, peptides and, more importantly, proteins. Although the classic semi-empirical work of Hammett remains relevant and is used for pK_a_ prediction in small molecules [22], in peptides and proteins pK_a_ prediction is dominated by the use of models that are both semi-empirical and computationally demanding, and statistical models that need large data sets for training [23].

The work of Chiang and Tai [8] provided, for the first time, an *ab initio* method to calculate a quantity (the Inductive Index) that correlated very well with ionization constants of a wide array of compounds and also with a range of observed chemical properties. It was also a crucial first step, from a theoretical standpoint, to discriminate the effect of one chemical group or moiety on another in terms of its inductive (that is, through-bond) and field (through “space”) components, which are generally conflated [24]. The Inductive Index algorithm has several relevant properties: i) it allows calculation of non-aromatic molecules of, in principle, any size, ii) it is a simple *ab initio* method that only requires knowledge of molecular covalent structure, electronegativities and bond lengths, iii) it considers formal charges present, iv) by definition field effects are not incorporated and, v) on a given bond, the algorithmic representation of the effect of all other bonds within the molecule is unique, save in trivial cases such as, for example, geometric (*cis/trans*) isomers.

Chiang and Tai's original approximation, however, has important limitations that require further discussion. First, electronegativity as a central chemical concept has been developed considerably since Pauling's seminal contributions [25]. The main limitation of Pauling's formulation of electronegativity -used by Chiang and Tai-is that it is conceived as a constant atomic property, not affected by, for example, the state of hybridization of electron orbitals or the covalent structure of the molecule [9]. Second, Chiang and Tai's method is unclear on how to calculate Inductive Indexes in cyclic molecules, especially those that contain more than one ionizable group. Third, it does not provide a method for calculation of Inductive Indexes in aromatic molecules, as explicitly stated by the authors. Fourth, the treatment of the electronegativity of charged atoms follows an *ad hoc* rule originally proposed by Pauling [25], regarding their apparent change of electronegativity. Fifth, while the general correlation of their Inductive Index values with ionization constants is remarkable for a wide range of pK_a_ values and compounds, it decreases when a more limited pH range is used, that is, it reflects subtler differences in molecular structure less accurately. Finally, the method is very useful for comparison of identical chemical groups (e.g. primary amines, carboxylic acids) but does not perform as well when comparing similar but not identical groups (e.g. primary and secondary amines).

The simplicity and applicability of Chiang and Tai's calculation method made it a very attractive initial approach to give “inductive effects” a numerical measure that could be contrasted to quantitative experimental data. We soon found, however, that correlation between Chiang and Tai's Inductive Index (calculated on C_α_) and measured pK_a_ values, while suggestive, was far from satisfactory (R^2^ = 0.55). We therefore modified their specific method (while retaining their general formal approach) to the calculation of Inductive Indexes (for a detailed discussion of our modifications see Supplementary Information).

*Ceteris paribus*, the distinctive properties of a chemical group are determined by two factors, namely, the nature of the group (e.g. amino, carboxyl, thiol) and the effects -inductive or field-of linked moieties. Specifically, the measured differences of pK_a_ of amino acid α-groups, in our interpretation, are a function of the inductive effects exerted by the different side-chains and reflect their electron donating or withdrawing power. Therefore a good correlation between *ii* (calculated on C_α_) and pK_a_ of α-groups gives us a quantitative way to assess the relative electron withdrawing or donating power of the side-chains. Fig. 2 shows that the correlation between our Inductive Index and α-amino pK_a_ is very significant, demonstrating that the modified Chiang and Tai algorithm can be usefully extended to amino acids.

Since bond polarization affects all neighboring groups, the state or nature of α-groups should affect side-chain properties. Therefore, we studied whether Inductive Index calculations correlated with ionization (pK_a_) of side-chains when the α-groups were covalently modified. We tested four ionizable amino acids (Asp, Glu, His and Cys) with and without α-group modifications (Fig. 3). The correlations between the pK_a_ of ionizable side-chains with Inductive Index values were very significant. As predicted by the calculations, in all cases removal of a negative charge by modification of the α-carboxyl group (while retaining an strongly electron-withdrawing protonated α-amino) yielded the lowest pK_a_. Conversely, removal of the protonated α-amino group by N-acetylation (while preserving a strongly electron-donating deprotonated α-carboxyl) yielded the highest pK_a_ in all cases. It is important to note that for equal structure the values calculated are equal (Table 3), but nonetheless correlated extremely well with changes in the pK_a_ of *very different chemical groups* (carboxyl, thiol and imidazole) in widely different ranges of pH. It is also of note that the response (slope) of each ionizable group to inductive input is characteristic. This property probably reflects both the polarizability of the ionizable group (greatest for the thiol group in cysteine) and the number of bonds between the ionizable group and the backbone (the slope is almost twice for the β-carboxyl of aspartic acid than for the γ-carboxyl of glutamic acid), independently supporting our choice of transmissivity factor (1/α).

### 4.2 Inductive index and peptides

Having found significant correlations between Inductive Index values and ionization constants (as pK_a_) in amino acids, it was natural to explore whether these correlations extended to peptides, as one aim of this investigation was to extend the *ab initio* calculation of Inductive Indexes to peptides to establish whether they are useful to understand some of their properties. However, many peptides have a very limited solubility in water, especially if they are composed of relatively hydrophobic residues and termini are covalently modified, constraining our ability to perform accurate potentiometric titrations to determine ionization constants. To circumvent this experimental limitation, we took advantage of the well-established fact that the pK_a_ of α-groups in fluorescent amino acids can be determined by the changes in fluorescence upon titration [15,16]. The possible contribution of inductive effects to their fluorescence has not, however, been quantitatively examined. To ascertain if there is a correlation between Inductive Index and tryptophan fluorescence we used the same strategy of Fig. 3, that is, we measured the Mean Fluorescence Lifetime (τ) in tryptophan as a zwitterion and in N- and C-covalently modified derivatives (N-acetyl-Trp, Trp-amide and N-acetyl-Trp-amide, Fig. 4). The correlation with Inductive Index values (which were the same calculated for identically modified amino acids with ionizable side-chains) was very significant (R^2^ = 0.9678), indicating that electron donating -or withdrawing-groups, and therefore bond polarization, determine τ to a significant extent at the amino acid level.

We then calculated Inductive Indexes on the C_α_ of tryptophan in a series of peptides to determine whether our observations at the amino acid level were applicable to larger, and more relevant, molecules. First, we chose a series of dipeptides and changed the amino acid adjacent to Trp (WA, WV, WM and WN). All peptides were N-acetylated and C-amidated for two reasons: i) to avoid a pH dependence of fluorescence and ii) to determine whether the inductive contribution of the side-chain of an adjacent amino acid sufficed to measurably change the fluorescence response of the Trp residue. Our results show that this is clearly the case, as shown in Fig. 5 (bottom panel). It is important to add that, in the case of the dipeptides, the only difference in *ii* value on the C_α_ of tryptophan is due to the side-chain of the amino acid being varied since all other factors are constant. Therefore, the graph of *ii* value of each side-chain (A, V, M and N, Table 2) against τ should give an identical correlation to that shown in Fig. 5, which is indeed the case.

We next studied the effect of distance, in number of residues, from the amino acid being varied on the fluorescence of the Trp residue. To this end we designed a series of tetrapeptides in which either electron-donating (Asp^−^) or withdrawing (Asn) residues were progressively moved away from the Trp residue (GGWX, GWGX and WGGX, where X is either Asp or Asn). The correlations for these tetrapeptides were also very significant as shown in Fig. 5 (bottom panel). For comparison purposes, the peptide GGWG was measured and included in the correlation of all the peptides against our calculations, which was also significant as shown in Fig. 5 (top panel).

Taken together, our results show a significant correlation between Inductive Index, a magnitude calculated solely from covalent structure and electronegativities, and two *independent* properties of amino acid side-chains: pK_a_ of ionizable groups and fluorescence lifetime of the indole group of tryptophan.

## 5 Conclusions

The successful prediction of ionization constants of functional groups of biomolecules, small and large, remains one of the most important areas of biochemical interest, both from basic and applied perspectives [26,27]. Currently it is generally accepted that, in the case of proteins, the two key amino acid properties studied here, ionization (as pK_a_) and Trp fluorescence (as τ), are largely determined by the specific environments generated by the three dimensional structure the protein acquires in the folding process. It is reasonable to suppose, however, that other factors, hitherto relatively overlooked, exert significant influence over them. One such factor, as our results show for Trp fluorescence, is the propagation of polarization of bonds in groups of atoms of different electronegativity, which in the context of the constant backbone structure of peptides (and proteins) are located on side-chains.

Although our modified CT (MCT) algorithm is not a pK_a_ predicting algorithm *per se*, the fact that there is a linear relation between pK_a_ and Trp fluorescence with Inductive Index values implies that the latter capture an intrinsic property of molecules in terms of two of the most basic chemical parameters, namely, covalent structure and electronegativity. Inductive Index calculations are simple and straightforward; they require neither steric arguments nor the use of statistical models that rely on many observations/data points for training.

If inductive effects through the peptide bond are, as our study suggests, a factor to take into account in understanding the complex behavior of functional groups in peptides, and maybe proteins, there are important implications for our understanding of the factors that determine the functionality of groups. For example, active sites of enzymes or binding sites of peptide/protein ligands, many of which are highly pH dependent. To illustrate, Fig. 3 shows that the thiol group of cysteine is very sensitive to inductive effects, a fact that may comport consequences, for example, for the catalytic properties of cysteine proteinases or the formation of disulfide bonds during oxidative folding.

Polarization of the electron clouds of side-chain functional groups is a consequence of the inductive effects of the rest of the molecule (α-groups in individual amino acids, or the sum of side-chains and backbone elements in peptides). Consequently, our results show that the amino acid sequence of a peptide determines a set of initial physicochemical conditions -independent of steric effects and microenvironments-that have a direct relation to the functional group.

Our results suggest that the inductive contributions from the amino acid sequence of a protein modify the density of the electron cloud of the atoms responsible for the formation of intramolecular hydrogen bonding, in a way that is particular to each atom depending on the sequence of neighboring amino acids. This could have further implications on backbone-based models of protein folding, such as the one developed by Rose et al. [28].

### 5.1 Necessary refinements and future directions

The value of the transmission factor is a heuristically selected number. We believe that α is likely different for every bond and probably a function α(χ_a_, χ_b_) depending on the electronegativity of the bonded atoms and the nature of the bond (as considered in the Hinze & Jaffe electronegativity scale). Nonetheless, our selected transmission factor constitutes an average that seems to capture the overall effect of distance on inductive polarization but a more detailed exploration is clearly necessary to provide a more accurate method of calculation and, eventually, a more complete explanatory theory.

Chiang and Tai stated that their method of calculation did not include aromatic molecules. It is important to extend the capacity of the modified algorithm to incorporate aromatic groups. The Inductive Index value for aromatic amino acid side-chains on their C_α_, and therefore on vicinal amino acids, can as of now be obtained by interpolation of their measured α-amino pK_a_ in Fig. 2.

Proline presents a particular calculational challenge for two reasons: i) it is not clear to us at this point how to adapt the algorithm to accurately reflect its cyclic structure, (i.e. it does not have a basic-structure/substituent form like the α-amino acids) and, ii) the pK_a_ values of its ionizable groups are not proportional, as is the case of all non-ionizable α-amino acids.

## CRediT authorship contribution statement

**Jesús Lara-Popoca:** Software, Investigation, Methodology, Visualization, Writing - original draft. **Henrik S. Thoke:** Investigation, Methodology. **Roberto P. Stock:** Conceptualization, Methodology, Investigation, Resources, Supervision, Visualization, Writing - original draft, Writing - review & editing. **Enrique Rudino-Pinera:** Funding acquisition, Resources. **Luis A. Bagatolli:** Resources, Methodology, Supervision, Writing - review & editing.

## Declaration of competing interest

No interests declared.
