# Supplementary material for: Inductive effects in amino acids and peptides: Ionization constants and tryptophan fluorescence
Source: Biochem Biophys Rep. 2020 Sep 13;24:100802. doi: 10.1016/j.bbrep.2020.100802 (PMC7498751; doi:10.1016/j.bbrep.2020.100802)
Supplement: Multimedia component 1 [file mmc1.docx]

**Supplementary Information**

**Inductive effects in amino acids and peptides: ionization constants and tryptophan fluorescence.**

Lara-Popoca, Jesusa╪ ; Thoke, Henrik S.b ╪; Stock, Roberto P.a; Rudino-Pinera, Enriquea; Bagatolli, Luis A.b,c,*

**^a^**Instituto de Biotecnología, Universidad Nacional Autónoma de México, Departamento de Medicina Molecular y Bioprocesos, Av. Universidad #2001, Col. Chamilpa C.P. 62210, Cuernavaca, Morelos, México,

**^b^**MEMPHYS - International and interdisciplinary research network, Odense, Denmark.

**^c^**Instituto de Investigación Médica Mercedes y Martín Ferreyra (INIMEC-CONICET-Universidad Nacional de Córdoba), Friuli 2434, 5016- Córdoba, Argentina,

^╪^ These authors contributed equally to this work.

* [lbagatolli@immf.uncor.edu](mailto:lbagatolli@immf.uncor.edu)

**Our method of calculation, value selection, calibration and heuristics.**

Considering i) the scarcity of non-statistical theoretical methods to estimate the importance of inductive effects on various (bio)chemical properties of amino acids and peptides (e.g. pK_a_ of ionizable groups) and, ii) the jump in complexity from dealing with small organic molecules or single amino acids to peptides/proteins, we opted for an heuristic approach for refining the original Chiang and Tai (CT) algorithm. This meant that from experimental data and a first round of calculations, we systematically modified one parameter at a time (e.g. different electronegativity scales, different values for transmission factor 1/α, considering all bond lengths equal to 1, etc.), while staying well within theoretically sound boundaries. After obtaining a better correlation between experimental data and calculations, we then proceeded to refine the model by modifying a different parameter.

Our first choice of parameter to modify was electronegativity. Pauling's formulation of electronegativity was carefully critiqued very early on, most notably by Mulliken, who pointed out serious limitations, particularly its unitless nature, and proposed an alternative conceptualization [1, 2], which has since become known as absolute electronegativity. Starting from Mulliken's electronegativity, Hinze and Jaffé [3] extended the general concept of electronegativity of an atom to that of electronegativity of orbitals and their state of hybridization. Considering the limited number of atomic species in amino acids and peptides, it added a level of subtlety that was of particular interest to our study. More specifically, it allowed us to explore the effects of small differences in electronegativity that in Pauling and Mulliken's scales would be otherwise indistinguishable because the differences in electronic structure of the bonds cannot be incorporated; they provide only an invariant electronegativity value for each atom.

With these considerations in mind, we derived some insights from our experimental results to guide our selection.

The scale of orbital/bond electronegativity of Hinze and Jaffe (*HJ*) introduced a level of refinement into the calculation of CT polarity indexes. However, these refinements were insufficient for us to effectively address two important cases: that of serine and threonine on one hand, and alanine and valine on the other. If we consider the two simplest side-chains, namely glycine and alanine (α-amino pK_a_ of 9.67 and 9.80, respectively), it is evident that the inductive effect is exerted directly on the C_α_ and it is not clear what other structural features would be of relevance to explain their differences in pK_a_ values. If our hypothesis is correct, it follows that the electron donating power of the methyl group is greater than that of the single hydrogen. However, the cases of serine and threonine (α-amino pK_a_ of 9.11 and 9.01, respectively), and of alanine and valine (α-amino pK_a_ of 9.80 and 9.55, respectively), consistently indicate that substitution of hydrogen(s) by methyl group(s) *decreases* the pK_a_ of the amino group (and therefore of the carboxyl group, Figure 1), in apparent contradiction to the reference case of glycine and alanine. However, what is consistent in these two cases is that hydrogens are removed from C_β_ and a CH_2_ or CH_3_ become a CH. This suggests that in a X_2_CH-CH_3_ group, the carbon of the X_2_CH has a weaker pull on the electron cloud than the carbon on the CH_3_ group. This situation translates into a lower apparent (relative) value of electronegativity for the carbon atom in a X_2_CH group than that of a carbon in a CH_3_ -or even a CH_2_- group. This observation suggested a further refinement of the electronegativity values that we used, which were not present in the original *HJ* scale. These added values are consistent with qualitative approximations of inductive contributions of substituent groups put forth by March [4]. The final values of electronegativity used for all calculations are summarized in Table S1.

| **Electronegativity Scales** | | | |
| --- | --- | --- | --- |
| **Atom** | **Pauling** | **Hinze-Jaffe (HJ)** | **Modified HJ** |
| C tetrahedral | 2.48 | 15.95 | 15.95 |
| C trigonal |  |  | 11.19 |
| C in CH_3_ |  |  | 17 |
| C in CH_2_ |  |  | 14.5 |
| C in CH |  |  | 13.6 |
| O tetrahedral | 5.39 | 30.5 | 30.5 |
| O tri (tri pi) |  | 20.17 | 20.17 |
| O- |  |  | 14 |
| N tetrahedral | 3.04 | 23.08 | 23.08 |
| N secondary |  |  | 35 |
| N+ |  |  | 24 |
| H | 2.21 | 14.34 | 14.34 |
| S tetrahedral | 3.21 | 20.27 | 20.27 |
| S secondary |  |  | 17 |
| S- |  |  | 25 |

**Table S1.** Electronegativities used to calculate all Inductive Indexes of side-chains of each non-aromatic amino acid and backbone elements (in peptides). Absent values in the Hinze-Jaffé column were not provided by the authors and were those effectively modified by us.

Our second choice of parameter to work on was bond length. How bond length modulates electronegativity differences of atoms [5] is a very relevant question. When adjusting our calculations using the modified *HJ* scale of electronegativity, we soon observed that general agreement between experimental data and the new Inductive Index calculations improved when bond distances were not included. Therefore, we maintained Chiang and Tai's concept of weighed difference of electronegativity (*δ_XY_*) but did away altogether with their concept of intensity of polarity $\left( \frac{\delta_{XY}}{r_{XY}} \right)$.

The third parameter we evaluated was the *transmission factor* $\left( \frac{1}{\alpha} \right)$. Since Hammett and Taft, it has been clear that the effect of any substituent on a reporter group decreases with distance (in number of bonds). Chiang and Tai implemented the decay of the effect as an exponential function of the number of intervening bonds (bond order, *n*). The choice of value for α must be within reasonable bounds, i.e., greater than 1 (since 1 would mean no attenuation with distance) but not so high that it will obliterate any differences in index of polarity of near bonds. However, it is not at all clear that all bonds would attenuate the effect of polarity differences equally. Therefore, a single value of $\left( \frac{1}{\alpha} \right)$ would act as an average for all attenuations at work in a molecule. Chiang and Tai recognized this quantity as a somewhat arbitrary value that they set to 2.7 following discussions in the literature of their day [6]. They used a heuristic approach: it was justifiable by the fact that it gave the best fit of their Inductive Index to the data. In our modified version, a value of α=1.9 consistently improved all correlations between calculation and experimental data and we therefore fixed this as the value when making all calculations at the amino acid level and it was subsequently kept for peptides.

The final parameter we examined was the value of the formal charges. We kept the formal treatment used by Chiang and Tai (without the division by bond length) but found that, when comparing amino acid side-chains with and without charged groups, the values used by them introduced large and consistent deviations from the best fit in the correlation between Inductive Index and α-amino pK_a_ (Figure 2). Considering that bond orders for charged atoms are difficult to assign (for example, the positive charge of a protonated amino group can be conceivably distributed between the nitrogen and its hydrogens, or unequally distributed in the resonating guanidinium in Arg, in both cases changing the bond order and affecting the calculations), we decided to keep the order constant but examine alternative values for both the positive and negative charges and their effect on the overall fit of the pK_a_ versus Inductive Index for all amino acids with charged side-chains. The values that gave the best agreement were +1.2 on charged nitrogens (in Arg, Lys and α-amino groups) and -0.9 for charged oxygens (Asp, Glu and α-carboxyl groups) and sulfur (Cys).

Importantly, all modified parameters were subsequently maintained for all calculations, namely, the effects of α-groups on side-chain pK_a_ (Figure 3) and tryptophan fluorescence at the amino acid level (Figure 4) and in peptides (Figure 5).

The final values for all calculations for backbone elements (excluding side-chains, which are operated separately considering their bond orders and are presented in Table 2) are summarized in the Table S2.

| ***ii* contributions for calculation** | |
| --- | --- |
| **Backbone** | ***ii* (x 10^3^)** |
| Central | 186.6 |
| Neighbor (left) | -357.4 |
| Neighbor (right) | 588.6 |
| **Terminal amino acid** |  |
| COO- (left) | -513.5 |
| COOH (left) | -312.1 |
| C-amide (left) | -333.5 |
| NH_3_^+^ (right) | 968.4 |
| NH_2_ (right) | 556.6 |
| N-acetyl (right) | 567.1 |
| **Peptide bond** |  |
| Left | -347 |
| Right | 347 |

**Table S2.** Values used for calculation of inductive indexes on the C_α_ of tryptophan in peptides. The contribution of the backbone elements of Trp itself is “Central”. The contribution of backbone elements to both left and right of Trp which are not terminal groups are defined as “Neighbors”. The contributions of the different terminal amino acids with their specified end groups are defined as “Terminal amino acid”. Right and left specifically refer to the direction in which the bond order of the group being considered decreases. For example, the effect of a protonated terminal amino group is always calculated to the “right” or, using the diagram for Chiang and Tai´s method (see Material and Methods), the contribution of all atoms of the molecule on atom A would be calculated to the “left”.

We present two calculations using our modified version of the CT algorithm to exemplify how we obtained the values for the entries in Tables 2 and 3: the valine side-chain and a backbone structure (N-Acetylated and with an unmodified ionized carboxyl α-group). It is worth noting that, for side-chains (Table 2), backbones (Table 3) as well as peptides (Table 4), all calculations were done considering the α-carbon as our “point of observation”, and then assigning the respective bond orders accordingly. For the valine side-chain, the calculation is:

$i=\frac{1}{\alpha}\delta_{CH}+\left( \frac{1}{\alpha} \right)^{2}\left( 6\delta_{{CH}_{3}} \right)$ [1S]

where $\delta_{CH}=\frac{\left( \chi_{H}-\chi_{C} \right)}{\left( \chi_{H}+\chi_{C} \right)}$ , and χ_C_ is the electronegativity corresponding to a carbon in a *CH* group (see table S1). This is multiplied by 1/α because the bond is of order 1. Notice that there are no bonds of order 0 since the carbon-carbon bond cancels out in the calculations. Next, for the methyl groups we have two sets of three $\delta_{{CH}_{3}}=\frac{\left( \chi_{H}-\chi_{C} \right)}{\left( \chi_{H}+\chi_{C} \right)}$ , where χ_C_ is the electronegativity of a carbon in a CH_3_ group, and these bonds are of order 2, hence we multiply by $\left( \frac{1}{\alpha} \right)^{2}$.

For the backbone structure, the calculation is as follows:

$i=\left( \delta_{C_{\alpha}H}+\delta_{C_{\alpha}N} \right)+\left( \frac{1}{\alpha} \right)\left( 3\delta_{C=O^{-}}+2\delta_{{NH}_{2}}+\delta_{NC} \right)+\left( \frac{1}{\alpha} \right)^{2}\left( 2\delta_{C=O} \right)+\left( \frac{1}{\alpha} \right)^{3}\left( 3\delta_{{CH}_{3}} \right)+\left( \frac{1}{\alpha} \right)\left( -0.9 \right)$ [2S]

The electronegativities for *δC=Oˉ* are for a trigonal carbon and an oxygen with a negative charge (C tri and O- respectively in Table S1).

| **Mean Fluorescence Lifetime Data** | | | | |
| --- | --- | --- | --- | --- |
| **Peptide** | **τ (ns)** | **χ^2^** | **STE (ns)** | **Width** |
| Tryptophan | 2.54 | 1.35 | 0.01 | 0.58 |
| NATA | 2.71 | 0.35 | 0.01 | 0.11 |
| Tryptophan Amide | 1.44* | 0.06 | 0.05 | 0.49 |
| N-Ac-L-Trp | 4.24 | 0.33 | 0.02 | 0.62 |
| WGGD- | 1.82 | 1.13 | 0.03 | 0.06 |
| GWGD- | 1.26 | 0.05 | 0.01 | 0.27 |
| GGWD- | 1.08 | 1.23 | 0.04 | 0 |
| WGGN | 1.80 | 1.05 | 0.01 | 0 |
| GWGN | 1.21 | 0.32 | 0.01 | 0.11 |
| GGWN | 0.95 | 0.71 | 0.01 | 0.10 |
| WN | 1.55 | 0.61 | 0.01 | 0.22 |
| WM | 1.71 | 0.34 | 0.01 | 0.47 |
| WV | 1.91 | 0.45 | 0.01 | 0.18 |
| WA | 1.82 | 0.18 | 0.01 | 0.31 |

**Table S3**. Mean Fluorescence Lifetime (τ). N-Ac indicates N-acetyl. NATA stands for N-acetyl-tryptophan-amide.

(*) indicates that two lifetimes were needed to fit the data. The value shown corresponds to the main fraction, see material and methods.

**References**

[1] R.S. Mulliken, A New Electroaffinity Scale; Together with Data on Valence States and on Valence Ionization Potentials and Electron Affinities, Journal of Chemical Physics 2(11) (1934) 782-793.

[2] R.S. Mulliken, Electronic Structures of Molecules XI. Electroaffinity, Molecular Orbitals and Dipole Moments., J. Chem. Phys. 3(9) (1935) 573–585.

[3] J. Hinze, H.H. Jaffé, Electronegativity. I. Orbital Electronegativity of Neutral Atoms, J. Am. Chem Soc. 84 (1961) 540-546.

[4] J. March, M.B. Smith, March’s Advanced Organic Chemistry. Reactions, Mechanisms and Structure, 6th ed., John Wiley & Sons New Jersey2007, p 20-21.

[5] W. J.R., J.W. Hall, Electronegativity: the distance factor, Journal of Molecular Structure 674(1-3) (2004) 29-32.

[6] G.E.K. Branch, M. Calvin, The Theory of Organic Chemistry, Prentice-Hall Inc., New York1941.
